# Having a Positive Relationship to Physical Activity: Basic Psychological Need Satisfaction and Age as Predictors for Students’ Enjoyment in Physical Education

**DOI:** 10.3390/sports9070090

**Published:** 2021-06-24

**Authors:** Sascha Leisterer, Leonie Gramlich

**Affiliations:** Faculty of Sports Science, Leipzig University, Jahnallee 59, 04109 Leipzig, Germany; leoniegramlich95@gmail.com

**Keywords:** adolescents, affective states, emotion, school, teaching

## Abstract

Physical activity is fundamental to prevent common illnesses in youth and research shows that students who perceive enjoyment in physical education (PE) have a more physically active lifestyle. This study aims to identify psychological antecedents of student enjoyment in PE. We addressed this by assessing aspects of self-determination theory (SDT), including the extent of autonomy-supportive teaching in PE (reported by teachers), the satisfaction of students’ basic psychological needs, and student age (reported by students), via questionnaires. Correlational and multiple linear regression analyses of the collected data of *N* = 170 students (*M* = 14.3 ± 2.20, 10–19 years of age) and *N* = 10 teachers show that autonomy support is related to autonomy satisfaction in students (*r* = 0.20, *p* < 0.01). In turn, student perception of autonomy correlates with competence (*r* = 0.64, *p* < 0.001) and belonging (*r* = 0.37, *p* < 0.001). All three basic psychological needs predict enjoyment in PE (*F*(1, 163) = 19.59, *R*^2^ = 0.68, *p* < 0.01). Additional analyses show that higher student age predicts a decrease in enjoyment (Δ*R*^2^ = 0.04, *B* = −0.73, *β* = −0.21, *p* < 0.01). Thus, student enjoyment in PE, as a foundation for a physically active lifestyle, can help to prevent common illnesses by satisfying basic psychological needs in PE.

## 1. Introduction

The way in which students experience physical education (PE) is decisive in determining whether physical activity becomes an integral part of their lifestyle in the long term. While long-term benefits are related to enjoyment in PE [1], how to foster enjoyment in PE remains poorly understood. Therefore, there is a need to investigate the determinants of enjoyment in PE.

In general, enjoyment in school declines [2] as well as enjoyment of PE, over the course of a student’s school career [3,4]. Accordingly, it is not surprising that physical activity among adolescents is declining and only 25% of minors meet the exercise recommendations of the World Health Organization (WHO) [5].

But what can teachers do to motivate children and young people to engage in PE so that they satisfy WHO recommendations for regular physical activity? This is where the importance of feelings experienced during PE comes into play. Positive emotions, such as pleasure, play a decisive role in developing long-term exercise habits [1,6]. In addition, there is evidence that in childhood and adolescence, the foundations are laid for developing a physically active lifestyle [7,8]. Recent findings show that the influence of the teacher’s support of student autonomy in PE affects the physical activity of students via the students’ intrinsic motivation [9]. Thus, the investigation of the determinants of positive emotions in PE, such as enjoyment, is warranted. In turn, empirical findings can be translated into practical implications for teaching, which may, if implemented consistently, lead to more enjoyment in PE.

### 1.1. Autonomy Support, Self-Determination Theory, and Enjoyment in PE

Teachers employ autonomy-supportive teaching styles to favour adaptive behavioural and affective outcomes (e.g., intrinsic motivation, well-being) in students [10,11]. Autonomy support in PE is characterized by teaching that provides for students, for example, freedom of choice, activities of interest, moments of individual engagement, and acknowledging their worries [10]. Additionally, autonomy-supportive teachers communicate using non-controlling and student-centred language (e.g., providing helpful feedback) [10]. Adopting these teaching styles, therefore, supports PE that focuses on student development and internalization of educational values and content (such as a physically active lifestyle), as proclaimed by self-determination theory (SDT) [11].

SDT assumes that a person’s intrinsic motivation and well-being are both affected by satisfying three basic psychological needs which are autonomy, competence, and social belonging [12]. In addition, enjoyment is an important component in the development of intrinsic motivation [12]. Based on SDT, it can be assumed that promoting these basic psychological needs in PE increases the intrinsic motivation and the well-being of learners [13,14,15]. Interestingly, enjoyment is considered as one among the six components contributing to well-being in school [16]. It is, therefore, reasonable to assume that heightened well-being is associated with greater enjoyment among learners. Furthermore, intrinsic motivation can be described as the enjoyment perceived upon action performed for its own sake [17], which suggests an association between intrinsically motivated action and enjoyment. This raises the question as to whether PE that focuses on promoting the three basic psychological needs, that is, autonomy, competence, and social belonging, leads to more enjoyment among learners.

It has been shown that enjoyment is an integral part of PE [18] that may occur before (as joyful anticipation), during (as enjoyment while involved in a task), or after (joyful relaxation) PE [19]. Independent of its occurrence, enjoyment in PE has a positive core affect [20] characterized by pleasure, flow, and relaxation [19]. In other words, students perceive pleasure when anticipating PE, experience flow while involved in a PE task, and relax following a PE class perceived as joyful. Thus, enjoyment in PE represents a positive affective state that can enhance student well-being and engagement in sport and physical activity before, while, and after PE.

### 1.2. Empirical Findings Related to Enjoyment in PE

Although student enjoyment in PE appears to be beneficial, schools cannot maintain the initial euphoria of younger students towards learning when they reach higher grade levels [21]. Students’ positive emotions associated with school begin to decrease as early as elementary school [2]. Enjoyment in PE abates especially in early adolescence, as shown in longitudinal analyses [4]. Consequently, the challenge is to create an environment in PE for older children and adolescents that fosters enjoyment.

Autonomy-supportive PE has been shown to enhance student enjoyment in an educational setting. Several studies present correlations of task orientation in class, as well as perceived autonomy and competence, with enjoyment in female students [22,23] and student athletes [24]. Recently, it has been presented that autonomy-supportive teaching can improve student self-efficacy and intrinsic value towards PE, which reduces anxiety and fosters enjoyment, respectively [25]. Specific teaching methods (e.g., task orientation using the TARGET approach) can influence student perception of autonomy in PE and increase positive affective outcomes [26]. Furthermore, autonomy-supportive PE not only enables the need for autonomy but also for competence and for social belonging; satisfying all three basic psychological needs implies enjoyment in PE.

The three basic psychological needs for autonomy, competence, and social belonging, as well as intrinsic motivation, are predictors of affective states in PE. Intrinsic motivation, which is dependent upon the three basic psychological needs, showed a strong positive correlation (*r* = 0.75, *p* < 0.05) with student enjoyment [27]. In the same study, autonomy was most strongly correlated with enjoyment, reporting *r* = 0.59, *p* < 0.05 [27]. Especially in lower grade level students, autonomy is associated with positive affective states such as enjoyment in PE [27,28]. Further studies assume that autonomy and competence are especially important for student enjoyment in PE [21,29]. Accordingly, teachers should stimulate student enjoyment not only by supporting autonomy, but also student perception of competence [29,30]. The higher the perception of competence, the more intense the enjoyment is in students [23]. However, enjoyment seems to be age-dependent whereby studies show either a decrease [23] or an increase, especially in females [31], in enjoyment, as students become older. Social belonging is also considered an important predictor of enjoyment in PE, together with feelings of competence [32]. In contrast, other studies show that the basic psychological needs for competence and social belonging have a strong significant positive influence on enjoyment, whereas autonomy does not [33].

### 1.3. The Present Study

Research is needed on the topic of enjoyment in PE and its precise relationship to perceived competence, autonomy, and social belonging in students. In particular, the development of enjoyment in PE related to basic psychological need fulfillment throughout the years needs to be investigated. Also, research still focuses on deciphering which of the basic psychological needs influence enjoyment and to what degree. The aim of the present study is to analyze the role of the three basic psychological needs based on autonomy-supportive teaching regarding student enjoyment in PE to provide empirical insights for PE teaching. Thus, we will examine the question: To which extent can the satisfaction of basic psychological needs predict enjoyment in PE with special regard to student age? Referring to this question, we hypothesize that:

**Hypothesis** **1** **(H1).** *The higher the satisfaction of competence or autonomy or belonging is, the higher enjoyment is in PE.*

**Hypothesis** **2** **(H2).** *The older a student is, the lower the enjoyment is in PE.*

**Hypothesis** **3** **(H3).** *The three basic psychological needs and student age predict enjoyment in PE.*

## 2. Methods

### 2.1. Participants

The study involved *N* = 170 voluntary participants including both male (32.40%) and female (67.60%) students with an average age of *M* = 14.30 years (*SD* = 2.20, age range: 10–19 years of age). Sample size was estimated in advance using G*Power [34], based on *f* = 0.12, *α* = 0.05, and a test power of 1 − *β* = 0.95 for linear multiple regressions. The resulting calculation recommended a sample size of 160 participants. Participants were distributed among a total of two different secondary schools in Germany. According to the Ethics Advisory Boards of the institutions, the present research study is regarded as ethically harmless, as no person-specific data was collected and no intervention was conducted (see also Section “Institutional Review Board Statement”); participants had to be healthy and had to sign an informed consent. All student participants—additionally their parents in the case of minor participants—and teacher participants had to sign a consent form. Two weeks before the study was conducted, students and their parents were informed by means of an information sheet about the study procedure, objectives of the study, and anonymous treatment of the data. Parents who agreed to their child’s participation in the survey, as well as students of legal age, signed a declaration of consent before data were collected. In addition, *N* = 10 PE teachers from the participating schools signed a declaration of consent and then completed a short questionnaire on the degree of autonomy support in their sports lessons.

### 2.2. Measurements

#### 2.2.1. Autonomy Support Assessment Sheet

In the absence of a standardized German assessment tool when collecting data, a short questionnaire was designed for teachers, based on the current literature regarding the practical implications for autonomy-supportive teaching [35,36,37], to determine the extent to which they promote student autonomy in PE. The questionnaire asks teachers to respond to six items (e.g., “I ask students for suggestions and I am willing to realize these in PE” or “I provide different options, so that the students can choose their tasks individually”) using a dichotomous “yes”–“no” scale to ascertain whether or not they employ autonomy-promoting measures in their lessons.

#### 2.2.2. Psychological Basic Need Satisfaction: PBNSS

The German version of the Psychological Basic Need Satisfaction Scale for Physical Exercise (PBNSS) [38] was used to measure the students’ basic psychological need satisfaction in PE. The PBNSS consists of three subscales each composed of three to four items (e.g., “I feel the way I exercise is an expression of myself”, “I feel confident in my ability to exercise regularly”, “I feel connected to the people I interact with while we exercise”), which measure the perceived autonomy, competence, and social belonging of students. Items were rated according to a 7-point Likert scale (“do not agree at all”–“very strongly agree”). To fit data collection in PE, questionnaire items were reformulated slightly so that the statements matched the PE context and were understandable for all students (e.g., “exercising” was adapted to “practicing” or “people” was adapted to “classmates”). The German version of the PBNSS was found to be a satisfyingly reliable (Cronbach’s *α* between 0.62 ≤ *α* ≤ 0.85; retest reliability between 0.54 ≤ *r* ≤ 0.64) and valid (factor validity: *Χ*^2^(33) = 66.68, Comparative Fit Index CFI = 0.95, Tucker-Lewis-Index TLI = 0.92, Root Mean Square Error of Approximation RMSEA = 0.07) questionnaire [38].

#### 2.2.3. Enjoyment in PE: FEFS-J

Student enjoyment in PE was recorded using the standardized questionnaire for enjoyment in PE in adolescents (FEFS-J) [19]. The questionnaire has a three-factor structure including subscales for pleasure, flow, and relaxation. Every subscale contains three items (e.g., “I enjoy PE”, “PE helps me to clear my mind”, “In PE I am excited about specific activities”) rated according to a 4-point Likert scale (“never”–“always”). An acceptable to good reliability for all three subscales has been reported (internal consistency: flow *α* = 65, *ω* = 0.72; relaxation *α* = 0.85, *ω* = 0.89; pleasure *α* = 0.86, *ω* = 0.91; retest reliability 0.70 ≤ *r* ≤ 0.81) [19]. The FEFS-J scale has a very good total reliability (*α* = 0.91, *ω* = 0.94) [19]. Factor analysis of this three-factor structure reported an acceptable factor validity (*Χ*^2^(24) = 125.48, CFI = 0.99, TLI = 0.99, RMSEA = 0.06) [19].

### 2.3. Procedures

After all participants (or their parents) signed consent forms, the PBNSS and the FEFS-J were handed out to the students during the last 15 min of a PE lesson. Meanwhile, teachers self-rated their PE lessons using the autonomy support assessment sheet. Before answering the questionnaires, students and teachers were given the opportunity to read the instructions carefully and to ask questions regarding the questionnaires. The time it took to complete the questionnaires varied from 5 to 15 min. Afterward, completed questionnaires and the autonomy support assessment sheets were collected in an envelope, separate from the consent forms, to ensure anonymity. This was done in the presence of all participants.

### 2.4. Data Analysis

Collected data were first checked for outliers and distribution characteristics. Then, the absolute subscore for competence was added to the subscore for social belonging and the subscore for autonomy to receive a total value, the basic psychological need satisfaction score (BNSS). The teachers’ autonomy support scores were summed up to an autonomy support index by deducting the summed scores of positive (“yes”) from negative answers (“no”), resulting in an index ranging from −6 to +6. Second, descriptive statistics were calculated to report means, standard deviations, and ranges. Third, correlations between age, the three basic psychological need scores of the PBNSS, and enjoyment scores of the FEFS-J were computed. Fourth, a multiple linear regression analysis was conducted to predict enjoyment in PE with stepwise inclusions of the assessed independent variables (i.e., competence, autonomy, belonging, age) in hierarchical order (i.e., from highest to lowest correlation). SPSS version 27 was used to analyse the data.

## 3. Results

### 3.1. Descriptive Statistics and Correlational Analyses

Table 1 shows the descriptive statistics of the variables for age, autonomy support index, basic psychological needs scores, and enjoyment in PE. Table 2 depicts Pearson’s *r* correlation values between the autonomy support index, student age, the three basic psychological need scores, and enjoyment in PE. The autonomy support index correlates significantly with student age (*r* = −0.25, *p* < 0.001), the basic psychological need for autonomy (*r* = 0.20, *p* < 0.01), and enjoyment in PE (*r* = 0.17, *p* < 0.05). Competence correlates significantly with autonomy (*r* = 0.64, *p* < 0.001), belonging (*r* = 0.99, *p* < 0.001), and enjoyment in PE (*r* = 0.76, *p* < 0.01). Autonomy correlates significantly with student age (*r* = −0.23, *p* < 0.01), belonging (*r* = 0.37, *p* < 0.001) and enjoyment in PE (*r* = 0.66, *p* < 0.01). Belonging correlates with enjoyment in PE (*r* = 0.46, *p* < 0.01). Student age correlates significantly with enjoyment in PE (*r* = −0.43, *p* < 0.01).

### 3.2. Stepwise Multiple Logistic Regression

According to the results of the correlational analyses, competence, autonomy, and belonging were included stepwise into the multiple regression analysis to predict enjoyment (Table 3). First, competence had the strongest significant correlation with enjoyment and was included in the regression model, resulting in Δ*R*^2^ = 0.48, *B* = 0.86, *β* = 0.48, *p* < 0.01. Second, autonomy had the second strongest correlation with enjoyment and was also included, resulting in Δ*R*^2^ = 0.05, *B* = 0.19, *β* = 0.13, *p* < 0.01. Third, belonging was included, resulting in Δ*R*^2^ = 0.01, *B* = 0.08, *β* = 0.13, *p* < 0.05. Fourth, student age was included, resulting in Δ*R*^2^ = 0.04, *B* = −0.73, *β* = −0.21, *p* < 0.01. Finally, the linear regression model with all four predictive variables (competence, autonomy, belonging, age) resulted in *F*(1, 163) = 19.59, *R*^2^ = 0.68, *p* < 0.01.

## 4. Discussion

Based on our results, the present study contributes empirical insights into autonomy-supportive teaching in PE that is related to the satisfaction of the three basic psychological needs as antecedents of student enjoyment in PE taking into account student age as a restriction to the described relationships. We first hypothesized that the higher the satisfaction of the need for competence, autonomy, or belonging is, the higher a student’s enjoyment is in PE. Our analyses reveal significant correlations between the three basic psychological needs and enjoyment in PE. Competence is strongly related, autonomy is moderately to strongly related, and belonging is moderately related to enjoyment in PE. Consequently, we accept our first hypothesis. Additionally, our analyses show that teacher self-reported autonomy support in PE is significantly related to autonomy satisfaction and enjoyment only. However, student self-reported autonomy is strongly related to competence and moderately related to belonging. Thus, we can assume that a change in the basic psychological need for autonomy via autonomy support may be related to a change in competence and belonging in PE students.

Second, we hypothesized that as students advance in age, their enjoyment in PE decreases. Analyses show a significant moderate negative correlation between age and enjoyment in PE. Thus, we accept our second hypothesis. The correlational analyses identify significant negative relationships between age and teacher self-reported autonomy support and student self-reported autonomy satisfaction. In conclusion, the older students get, fewer benefits of basic psychological need satisfaction can be observed. According to our correlational analyses, we assume that higher grade level classes in PE are less autonomy-supportive, and older PE students are less autonomy satisfied.

Third, we hypothesized that the three basic psychological needs and student age predict enjoyment in PE. The multiple linear regression analyses provide significant results that competence, autonomy, belonging, and age predict student enjoyment in PE. Up to 68% of the variances in student PE enjoyment can be explained via the satisfaction of the three basic psychological needs and student age. Thus, we accept our third hypothesis.

### 4.1. Enjoyment in PE: Favoring the Satisfaction of All Three Basic Psychological Needs over a Single One

In line with earlier research [4,21,28], our data show that student enjoyment in PE peaks in lower grades of PE and then declines as a student’s school career progresses. The question therefore arises, why do older students perceive less enjoyment and autonomy in PE? Although there is a paucity of research on factors influencing the decline of PE enjoyment during a student’s school career, it is likely that the answer lies in the fact that PE lessons become more output focussed in higher grade PE classes. In output-focussed higher grade PE classes, students are evaluated on their results, which in turn might favour a less autonomy-supportive and more control-based teaching styles, which may decrease student enjoyment.

In general, our analyses support current research regarding autonomy support, basic psychological need satisfaction, and enjoyment in PE. As shown in recent research [15,27], autonomy support in PE relates positively to student perception of autonomy and enjoyment. Additionally, we showed that autonomy support is related to the satisfaction of autonomy, which is associated with satisfaction of competence and belonging as well [15,24,39]. In conclusion, and in line with previous findings, autonomy support is positively related to the satisfaction of the three basic psychological needs, which in turn predicts enjoyment in PE [22,23,24,25,26].

Although satisfying the three basic psychological needs in PE predicts student enjoyment [26,32], the precise meaning of autonomy, competence, and social belonging for enjoyment in PE needs to be examined further. Several findings suggest that the satisfaction of competence is the most important predictor of enjoyment in PE [4,22] while others include the satisfaction of social belonging as a predictor as well [34]. Our regression coefficients can contribute to this discussion with a ranking of the relative importance of the three basic psychological needs in predicting student enjoyment. According to our results, while competence is most highly weighted, followed by autonomy and social belonging, the satisfaction of all three basic psychological needs combined predicts enjoyment whereby up to 64.00% of the variance of student enjoyment in PE can be explained. Therefore, we recommend that PE teachers employ measures that foster an environment where students can fulfil all three basic psychological needs together, instead of employing PE content that favours only one basic psychological need separately, for example, using cooperative games to satisfy social belonging and positively influence enjoyment in PE [40].

### 4.2. Methodological Strength and Limitations

A strength of this study is the randomly collected sample, which is not nested in PE classes, as is usually the case in school-based studies. In this way, we provide general insights into student PE enjoyment distributed throughout the age range of secondary school students. Yet, regression analyses are correlational analyses and no explanations of causality can be given here. Therefore, limitations in the interpretation of predictive relationships should be further investigated in intervention studies. In the absence of a standardized German questionnaire to assess teacher autonomy support in PE, when collecting the present data, this study is limited to teacher self-reported autonomy support. Future studies could use the lately validated German Multi-Dimensional Perceived Autonomy Support Scale for Physical Education [41], as shown recently [25].

### 4.3. Further Research

Future research in the field of autonomy support, SDT, and enjoyment in PE should address three important open questions related to this study. First, it remains unclear if control-based teaching styles contribute to a decrease in enjoyment in higher grade PE classes compared to lower grade PE classes with younger students. Thus, future studies should investigate longitudinal data regarding decreasing enjoyment of PE as students get older. Second, the investigation of causal relationships between autonomy support, basic psychological need satisfaction, and enjoyment in PE is necessary to derive empirically proven practical measures. In the future, quasi-experimental intervention studies are needed to better understand how to affect student enjoyment in PE in relation to the three basic psychological needs. Third, as this study’s analyses do not fully explain the variance of the variable enjoyment, further independent variables, for example, the attractiveness of a task [42], should be assessed to investigate further influences on PE enjoyment.

### 4.4. Practical Implications to Foster Enjoyment in PE

Based on our results, we recommend that PE teachers should support students by fostering their autonomy. This can be realized by adopting different autonomy-supportive teaching styles [11] or including targeted PE content designed to satisfy basic psychological needs [13,14,40]. In this way, teachers can help fulfil a student’s basic psychological needs for competence, autonomy, and social belonging, and increase their enjoyment in PE. Especially in higher grades, autonomy support should be emphasized to counterbalance more standardized PE classes, and to enhance student enjoyment in PE. Among the many long-term benefits of enhancing student enjoyment in PE, and in line with WHO recommendations, is the development of a physically active lifestyle.

## Figures and Tables

**Table 1:** Descriptive statistics (i.e., means and standard deviations, range) of the variables for age, autonomy support, basic psychological needs, and enjoyment in PE.

	Age in Years	Autonomy-Support Index ^1^	Subscores			Basic Psychological Need Satisfaction Score ^4^	Enjoyment ^5^
			Competence ^2^	Autonomy ^3^	Belonging ^2^		
Mean	14.30	4.78	21.13	11.31	21.42	53.87	18.70
Standard deviation	2.20	1.27	4.41	3.47	5.31	10.55	6.36
Ranges	10–19	2–6	5–28	3–20	4–28	14–76	2–30

^1^ Possible autonomy-support index range: (−6)–(+6); ^2^ Possible subscore range: 1–28; ^3^ Possible subscore range: 1–21; ^4^ Possible basic psychological need satisfaction score range: 3–78; ^5^ Possible enjoyment range: 0–36.

**Table 2:** Pearson’s *r* correlation values between age, autonomy support, basic psychological needs, and enjoyment in PE.

	Autonomy-Support Index	Competence Subscore	Autonomy Subscore	Belonging Subscore	Enjoyment
Age in years	−0.25 ***	0.05	−0.23 **	0.06	−0.43 **
Autonomy support index		0.11	0.20 **	0.10	0.17 *
Competence subscore			0.64 ***	0.99 ***	0.76 **
Autonomy subscore				0.37 ***	0.66 **
Belonging subscore					0.46 **

Significance values: * *p* < 0.05; ** *p* < 0.01; *** *p* ≤ 0.001.

**Table 3:** Stepwise multiple logistic regression analysis to predict enjoyment by competence, autonomy, belonging, and age.

Step		*F*	*df*1	*df*2	*R* ^2^	Δ*R*^2^	*B*	*SE B*	*β*	*p*
	Model	19.59	1	163	0.68					<0.01 **
	Constant					0.10	3.87	3.43		0.26
1	Competence					0.48	0.86	0.11	0.48	<0.01 **
2	Autonomy					0.05	0.59	0.13	0.23	<0.01 **
3	Belonging					0.01	0.19	0.08	0.13	<0.05 *
4	Age in years					0.04	−0.73	0.16	−0.21	<0.01 **

Notes: *F* = *F*-ratio; *df* = degrees of freedom; *R*^2^ = standardized residuals; *B* = regression coefficient; SE B = standard error of regression coefficient; *β* = standardized regression coefficient; *p* = significance values (* *p* < 0.05; ** *p* < 0.01).

## Data Availability

The first author can be asked for the original data set supporting the results of this study.
